# Citric Acid Production by *Aspergillus niger* Cultivated on *Parkia biglobosa* Fruit Pulp

**DOI:** 10.1155/2014/762021

**Published:** 2014-11-03

**Authors:** Helen Shnada Auta, Khadijat Toyin Abidoye, Hauwa Tahir, Aliyu Dabai Ibrahim, Sesan Abiodun Aransiola

**Affiliations:** ^1^Department of Microbiology, Federal University of Technology, Minna 290281, Nigeria; ^2^Department of Microbiology, Usmanu Danfodiyo University, Sokoto 840001, Nigeria; ^3^School of Science, Abubakar Tatari Polytechnic, Bauchi 740001, Nigeria

## Abstract

The study was conducted to investigate the potential of *Parkia biglobosa* fruit pulp as substrate for citric acid production by *Aspergillus niger*. Reducing sugar was estimated by 3,5-dinitrosalicylic acid and citric acid was estimated spectrophotometrically using pyridine-acetic anhydride methods. The studies revealed that production parameters (pH, inoculum size, substrate concentration, incubation temperature, and fermentation period) had profound effect on the amount of citric acid produced. The maximum yield was obtained at the pH of 2 with citric acid of 1.15 g/L and reducing sugar content of 0.541 mMol^−1^, 3% vegetative inoculum size with citric acid yield of 0.53 g/L and reducing sugar content of 8.87 mMol^−1^, 2% of the substrate concentration with citric acid yield of 0.83 g/L and reducing sugar content of 9.36 mMol^−1^, incubation temperature of 55°C with citric acid yield of 0.62 g/L and reducing sugar content of 8.37 mMol^−1^, and fermentation period of 5 days with citric acid yield of 0.61 g/L and reducing sugar content of 3.70 mMol^−1^. The results of this study are encouraging and suggest that *Parkia biglobosa* pulp can be harnessed at low concentration for large scale citric acid production.

## 1. Introduction

Citric acid is one of the most common products which have a never ending demand in the global market. It plays a pivotal role in food and beverage industries and pharmaceutical, chemical cosmetic, and other industries for applications such as acidulation, antioxidant, flavor, enhancement, preservation, and plasticization and as a synergistic agent. Citric acid fermentation is one of the primitive fermentations but still its production is increasing with passage of time. In 2007, its global production has exceeded 1.6 million tons [1].

One of the most important fungi used in industrial microbiology,* Aspergillus niger, *has been employed for many years for the commercial production of citric acid. However, the worldwide demand for citric acid is increasing faster than its production and more economical processes are required.* A. niger *is most commonly used for citric acid production. This is because of the fact that this organism has capacity to utilize varieties of substrates due to its well-developed enzymatic system [2]. Although* Aspergillus niger *is the traditional producer of citric acid, during the last 30 years the use of yeasts for citric acid fermentation processes has attracted the interest of researchers. Among the yeast species,* Yarrowia lipolytica *is known as a potential producer of citric acid [3].

*Parkia biglobosa* (African locust bean) is a leguminous forest crop that belongs to the family Mimosaceae which provides to the West African population a range of products used in food and industrial and traditional medicine [4]. It is rich in nutrients such as carbohydrate, proteins, carotenoids, ash, and fibre. The nutritional composition of the pulp is favourable to the growth of* Aspergillus niger*. The conditions especially favourable to the citric acid fermentation are low nitrogen supply, high concentration of sugar, and nitrogen supplied as ammonium salts rather than as nitrates. The fruit pulp of the African locust bean is sweet to the taste, which indicates the presence of natural sugars and thus a potential energy source. The attractive yellow colour indicates the presence of phytonutrients, possibly carotenoids, which are important precursors of retinol (vitamin A). It has a sour taste which indicates the presence of ascorbic acid (vitamin C) [5]. Carbohydrate content was found to be 67.30%. This is much higher than the seeds (49.49%) as reported by Fetuga et al. [6] and is in agreement with the findings of Uwaegbute [7] that the fruit pulp contains more carbohydrates than the seeds. It is also higher than most legume seeds with only lentils and Bambara groundnuts coming close with a value of 65.0% [8]. Though proteins and fats also provide energy, carbohydrates are much cheaper and more easily digested and absorbed [9]. With this content of carbohydrate the African locust bean fruit pulp is a potential good source of energy given the recommended daily energy intake [8].

Strains of* Aspergillus niger* need a fairly higher initial sugar concentration in the medium [10]. The* Parkia biglobosa* pulp powder has high sugar content and this makes it suitable for the growth of* Aspergillus niger*. A high nitrogen concentration increases the growth of fungi and the consumption of sugars but decreases the amount of citric acid produced because it is a limiting factor in citric acid production [11]. Low level of phosphate favors citric acid production, however, the presence of excess phosphate has been shown to lead to the formation of certain sugar acids, decrease in the formation of carbon dioxide and the stimulation of growth [12].

The main interest of this study is to use* Parkia biglobosa *fruit pulp as a substrate for the production of citric acid. This is in order to explore its potentiality as a cheap raw material in the supply of citric acid for industrial usage and economic development.

## 2. Materials and Methods

### 2.1. Sample Collection

Two cups of* Parkia biglobosa *fruit pulp was purchased from Kofar Doya, Central Market, Sokoto State, Nigeria, into a clean polythene bag. The pulp powder was passed through a sieve and this was done to remove unwanted particles that were present in it.

### 2.2. Microorganism Used

*Aspergillus niger *species was taken from the available stock culture in the Mycology Laboratory, Department of Biological Sciences, Usmanu Danfodiyo University, Sokoto.

### 2.3. Inoculum Preparation

The conidial inoculum was used in the present study; conidia from a 5-day-old culture were used for inoculum preparation. The fungi were identified using lactophenol cotton blue. Inoculum preparation was done as described by Jin-Woo [13].

### 2.4. Preliminary Screening

The isolate was screened qualitatively for citric acid production by plate method on czapeckdox agar containing Bromocresol green as indicator 1% at pH 6. The spore suspension of the isolate was spread on the surface of the medium plates and allowed to grow for 5 days. Yellow zones indicated citric acid production.

### 2.5. Preparation of Fermentation Media for Citric Acid Production

The fermentation medium containing* Parkia biglobosa *fruit pulp 250 g, NH_4_NO_3_ 10.0 g, KH_2_PO_4_ 4.0 g, MgSO_4_·7H_2_O 1.0 g, and CaCO_3_ 2.0 g was transferred to 1000 mL conical flask and using a measuring cylinder, sterile distilled water was used to make up the volume to 1000 mL. 100 mL of fermentation medium was transferred into 100 mL conical flasks (25 flasks) using a sterile measuring cylinder, cotton plugged, and corked with aluminium foil. The flasks were autoclaved at 121°C for 15 min after cooling at room temperature and seeded with 2 mL of inoculums.

### 2.6. Effects of Variables on Citric Acid Production

Effect of pH was investigated on citric acid production. The range of pH investigated was 2, 4, 6, 8, and 10. The pH was adjusted using 1 N HCl and 1 N NaOH and temperature was investigated from 25°C, 35°C, 45°C, 55°C, and 65°C. The citric acid production was studied at different fermentation periods such as days 5, 6, 7, 8, and 9. The total titratable acidity was also determined by 0.1 N NaOH. The effect of different concentrations (1%, 2%, 3%, 4%, and 5%) of the substrate was carried out and the effect of different inoculum sizes (1%, 2%, 3%, 4%, and 5%) of the substrate was also studied.

All experiments were incubated in an orbital shaker. Each assay was carried out in duplicate and the mean of the duplicate analysis was reported in each figure.

### 2.7. Citric Acid Assay

Reducing sugar was estimated by 3,5-dinitrosalicylic acid (DNS) method. Anhydrous citric acid was estimated based on the rapid method for the determination of citric acid using pyridine-acetic anhydride method of Marrier and Boulet [14] and Guebel and Torres Darias [15].

## 3. Results

**Figure 1:**
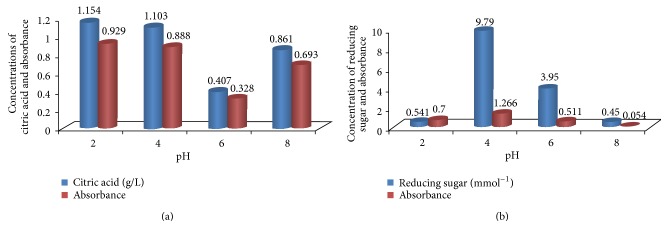
Effect of pH on citric acid production by* A. niger* cultured on* Parkia biglobosa* fruit pulp.

The utilization of* Parkia biglobosa *fruit pulp as substrate for citric acid production by* Aspergillus niger *was evaluated. And the effect of initial pH on citric acid production was investigated (Figure 1). The result showed that increase in pH brought about decrease in citric acid production. Maximum yield of 1.15 g/L was obtained at pH 2. Further increase in pH was found to decrease citric acid production.

The effect of different temperatures on citric acid production by the isolate was studied. A temperature of 55°C was found optimal for citric acid production as maximum citric acid of 0.62 g/L was produced at this temperature by the isolate. Further increase in temperature gradually decreased citric acid productivity by the isolate. The temperature of fermentation medium is one of the critical factors that have a profound effect on the production of citric acid.

The effect of vegetative inoculum size (1–5%) on citric acid production by* Aspergillus niger *(Figure 3) shows that the maximum citric acid production of 0.53 g/L was obtained with 3% inoculum size. As the inoculum size increased, citric acid production decreased.

The substrate concentration optima for citric acid accumulation by the isolates were studied and the result indicated that the maximum yield of the citric acid production was obtained at 2% of the substrate concentration with a value of 0.83 g/L and the reducing sugar content value of 9.34 mMol^−1^. The results for different substrate levels and their rate of citric acid and reducing sugar yields are shown in Figure 4.

The quantity of citric acid produced varies with both the type of microorganism and fermentation conditions. To determine the effect of fermentation period, fermentation was carried out for various time periods (5–9 days). In the current experiment, the rate of citric acid biosynthesis is shown in Figure 5. Production of citric acid increased with increase in the fermentation time. The maximum yield of citric acid produced was five (5) days after fermentation, Table 1. The optimal time of incubation for maximum citric acid production varies with both the organism and fermentation conditions. The rate of citric acid biosynthesis was studied and the maximum yield of citric acid (0.61 g/L) was after 5 days of fermentation

An illustration of the production of citric acid in the current study as compared to those produced in the literature is presented in Table 1.

## 4. Discussion

The production of citric acid by* Aspergillus niger* cultured on* Parkia biglobosa* fruit pulp showed that the highest yield (1.15 g/L) of citric acid was obtained at pH 2 and it declined as the pH increased from being acidic to alkaline (pH 8) with the yield of (0.86 g/L). This is due to the evidence of strong acidic conditions that are required for* Aspergillus niger *growth and production of citric acid. Similar observation has been noted by Guebel and Torres Darias [15]. This implies that using* Parkia biglobosa* fruit pulp as the source of carbon, the pH 2 is the best for the production of citric acid. It is worth noting that many publications have confirmed that the enhancement of citric acid production by* Yarrowia lipolytica* yeast strains occurs only in nitrogen-limited media in which pH is over 4.5 [16, 17]. Previous studies have also demonstrated a low-rate fed-batch and continuous production of citric acid under nitrogen-limiting conditions by the yeast* Candida lipolytica* or other* Candida* strains Kim et al. [18]; Tisnadjaja et al. [19]; Crolla and Kennedy [20]. About a four-stage process has been reported by Wieczorek and Brauer [21] for the continuous citric acid production using* A. niger* and recirculation of fermentation broth. The main actual problems at citric acid industry today are the still low productivities of discontinuous* A. niger* processes, requiring long operational times, higher investments, and production costs, compared with the novel yeast processes [22, 23]. Continuous operations for the production of citric acid by yeasts have increasingly received research interest in recent years. However, the yields were insufficient for an economically competitive industrial operation [23, 24]. Citric acid production takes places under growth limiting conditions [23].

**Figure 2:**
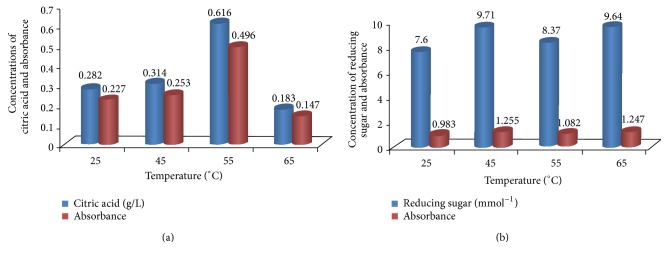
Effect of temperature on citric acid production by* A. niger* cultured on* Parkia biglobosa *fruit pulp.

**Figure 3:**
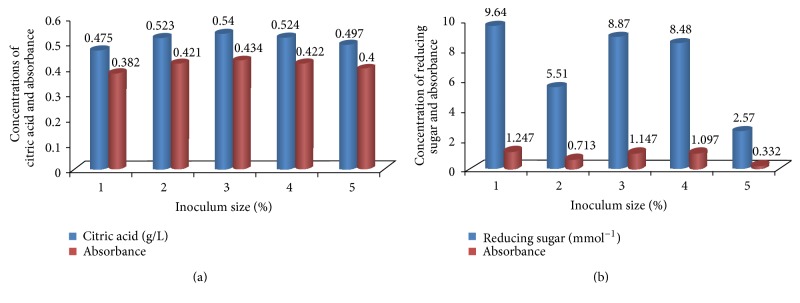
Effect of inoculum size on citric acid production by* A. niger* cultured on* Parkia biglobosa* fruit pulp.

**Figure 4:**
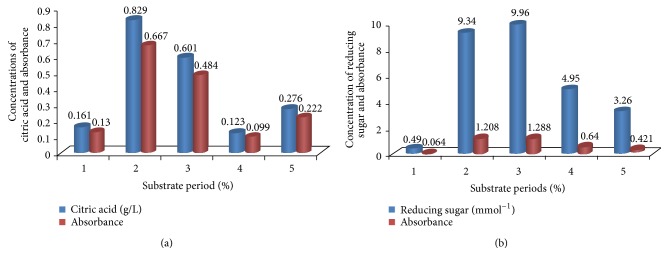
Effect of substrate concentration on citric acid production by* A. niger* cultured on* Parkia biglobosa* fruit pulp.

**Figure 5:**
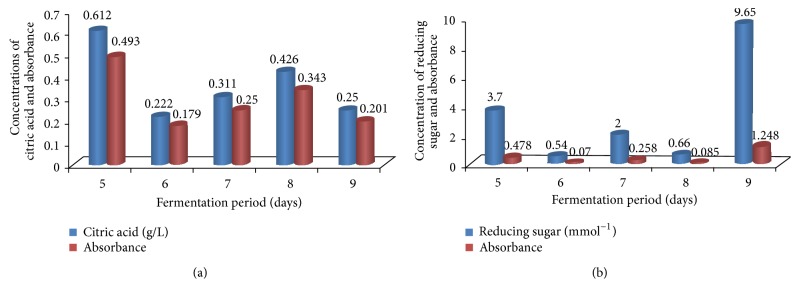
Effect of fermentation period on citric acid production by* A. niger* cultured on* Parkia biglobosa* fruit pulp.

The effect of temperature on citric acid production by* Aspergillus niger* cultured on* Parkia biglobosa *fruit pulp revealed that the maximum yield of citric acid (0.62 g/L) was obtained at an optimum temperature of 55°C (Figure 2). The high citric acid production was attributed to high enzymatic activity at higher temperatures. This report agrees with the findings of Kareem and Rahman [25]; they stated that the temperature of fermentation medium is one of the critical factors that have a profound effect on the production of citric acid. When the temperature of medium was low, the enzyme activity was also low, giving no impact on the citric acid production. But when the temperature of medium was increased above 55°C, the biosynthesis of citric acid decreased. It might be due to the accumulation of by-products such as oxalic acid. Different researchers have also used similar temperatures as the cultivation temperature and obtained higher values of actual product [26, 27]. This implies that* Parkia biglobosa* can support the biosynthesis of citric acid by* Aspergillus niger *at higher temperatures. This result is in contrast with that obtained by Kareem et al. [28] who recorded optimum temperature of 30°C for citric acid production by* A. niger* and Karthikeyan and SivaKumar [29], who recorded 28°C as optimum temperature for citric acid production by* A. niger* using banana peels as substrate.

Among the factors that determine the morphology and the general course of fungal fermentation, the type and size of inoculum are of prime importance. The present study revealed that maximum citric acid yield of 0.53 g/L was obtained with 3% of the inoculum size. This disagrees with the findings of van-Suijdam et al. [30], who reported 1.0% vegetative inoculum to be adequate for optimal production of citric acid. The citric acid yield was lower at 5% of the vegetative inoculum. This may be due to the clumping of* Aspergillus niger* at higher inoculum concentration. The increase in the number of inoculated spores primarily increases the level of acid production; however, on the long run this increase tends to force the process toward cellular crowding and to favor the consumption of sugar for biomass reproduction, thus resulting in a reduction in production of citric acid. The excessive reduction in the level of inoculation also causes prolongation in the adaptation phase, therefore decreasing an ideal outcome [31]. This result suggests that there appears to be an optimum amount of inoculum required for citric acid production.

Substrate concentration of the medium was discovered to have an immense effect on the rate of citric acid produced. 2% of the substrate concentration was discovered to give the maximum yield of 0.83 g/L for citric acid. This could be due to the fact that the substrate may contain sugars which inhibit citric acid production at higher concentrations. Hossain et al. [32] and Orthafer et al. [31] reported that some sugars such as galactose and arabinose inhibit citric acid production. This implies that the concentration of 2% of the substrate could support citric acid production but an increase from 2% would result in higher levels of certain sugars present in the pulp that are inhibitory to citric acid production. Interestingly, according to André et al. [16], a significant amount of lipids can be accumulated in shake-flask experiments under nitrogen-limited conditions during growth at a high glycerol concentration. The ATP lyase is likely to be induced under these conditions. Citric acid production has been a subject of interest for many workers, for example, Dhillon et al. [33]; Al-Khadir and Mohd [34]; Karthikeyan and Sivakumar [29]; Yadegary et al. [35]; Munshi et al. [36]; and Kareem and Rahman [25]. Different agroindustrial residues such as apple peel, banana peels, molasses, rice straw, jackfruit, pineapple wastes, pumpkin, sugarcane bagasse, apple pomace, and kiwi fruit peel have been investigated for their potential to be used as substrates for citric acid production using* A. niger.* High yields of citric acid have been produced by other organisms, especially* Yarrowia lipolytica* yeast,* Candida lipolytica, *and* Candida oleophila*, with a variety of substrates, for example, crude glycerol from biodiesel production, olive mill wastewater-based media,* n*-paraffins, and glucose Rywińska et al. [37]; Rywińska et al. [38]; Papanikolaou et al. [39]; Anastassiadis et al. [40]; and Crolla and Kennedy [20]. The volumetric citric acid productivity of the process with* Parkia biglobosa* fruit pulp is comparable to or lower than that reported by other authors who used such carbon substrates as coconut husk, molasses, whey/sucrose, and olive mill waste water (Table 1).

The optimal time of incubation for maximum citric acid production varies with both the organism and fermentation conditions. The rate of citric acid biosynthesis was studied and the maximum yield of citric acid (0.61 g/L) was produced after 5 days of fermentation. Extension of the fermentation period brought about depletion in the yield of citric acid produced. In batch-wise fermentation of citric acid, the production started after a lag phase of one day and reached maximum at the onset of stationary phase or later. It might be due to the decreased available nitrogen in fermentation medium, the age of fungi, and depletion of sugar contents. Similar type of work has also been reported by Wieczorek and Brauer [21]. Previous reports by van Suijdam et al. [30]; Chaudary et al. [41]; and Hang and Woodams [42] stated that much time causes the decrease of nitrogen and sugars in the substrate, thereby a reduction in citric acid production. This implies that the fermentation period used was suitable for the production of citric acid as higher yield is harvested at early stage thereby cutting down the cost needed to maintain the fermentation for longer time.

## 5. Conclusion

The study has revealed that* Aspergillus niger *can produce citric acid when cultured on* Parkia biglobosa *fruit pulp as source of carbon. The result of this study indicates that the use of* Parkia biglobosa *fruit pulp for fungal production of citric acid might represent an efficient method of cost reduction in the production and concomitantly producing organic acid of valuable importance.

## Figures and Tables

**Table 1:** Comparison of citric acid production between results in the current research and the literature values.

Organism	Substrate	Fermentation configuration	Citric acid yield (g/L)	Reference
*Aspergillus niger *	*Parkia biglobosa* fruit pulp	Shake flask	1.15	Present study
*Aspergillus niger *	Coconut husk	Shake flask	1.00	Sukesh et al. [43]
*Aspergillus niger *	Whey/sucrose	Shake flask	106.50	El-Holi and Al-Delaimy [44]
*Aspergillus niger *14/20	Pumpkin	Shake flask	10.35	Majumder et al. [45]
*Aspergillus niger *	Tapioca	Shake flask	1.60	Sukesh et al. [43]
*Aspergillus niger *	Apple	Shake flask	2.10	Sukesh et al. [43]
*Aspergillus niger* 14/20	Molasses	Shake flask	7.72	Majumder et al. [45]
*Aspergillus niger *	Bagasse	Shake flask	0.24	Vaishnavi et al. [46]
*Aspergillus niger *	Beet molasses	Shake flask	240.10	Lotfy et al. [47]
*Aspergillus niger *	Corn steep liquor	Shake flask	10.50	Lotfy et al. [47]
*Aspergillus niger* ATTCC 9142	Beet molasses	Shake flask	35.00	Roukas [48]
*Aspergillus niger* CBS733.83	Sugar cane bagasse	Shake flask	21.24	Pallares et al. [49]
*Yarrowia lipolytica *	Glycerol	Bioreactor	124.50	da Silva et al. [50]
*Yarrowia lipolytica *	Whey/sucrose	Shake flask	106.50	da Silva et al. [50]
*Yarrowia lipolytica *	Crude glycerol	Bioreactor	35.00	Papanikolaou et al. [51]
*Yarrowia lipolytica* IMUFRJ50682	Raw glycerol	Shake flask	12.94	Wojtatowicz et al. [52]
*Yarrowia lipolytica* ACA-DC50109	Olive mill waste/glucose	Shake flask	28.90	Papanikolaou et al. [39]
*Yarrowia lipolytica *	Olive mill waste water	Shake flask	18.1	Sarris et al. [53]
